# Low Mortality and Key Aspects of Delivery of Care for End-Stage Renal Disease in Italy

**DOI:** 10.1100/tsw.2009.43

**Published:** 2009-05-21

**Authors:** Alexander Lauder, Arrigo Schieppati, Ferruccio Conte, Giuseppe Remuzzi, Daniel Batlle

**Affiliations:** ^1^ Northwestern University Feinberg School of Medicine, Chicago, IL, USA; ^2^ Mario Negri Institute of Research, Bergamo, Italy; ^3^ A.O. Ospedale di Melegano, Milano, Italy

**Keywords:** public health/health policy, renal disease, clinical performance measures, mortality

## Abstract

End-stage renal disease (ESRD) is a global health problem. There are differences in mortality among patients with ESRD amid industrialized countries that may be related to their respective systems of delivery of care. A nationwide survey was completed in Italy, a country with low mortality rate for ESRD patients, in order to help understand key aspects of ESRD delivery of care that contribute to mortality. Survey responses were obtained and analyzed from 131 of 575 dialysis centers (23%), covering data from 13,170 dialysis patients in 2006. The mortality rate was 11.2% and the prevalence of diabetes-associated kidney disease was 21%. Of the patients, 88% were on hemodialysis and 12% were on peritoneal dialysis. Most patients were in the age range of 65–75 years (66.7%), were seen by a nephrologist at CKD stage 3, and began dialysis at mean estimated GFR of 9.6 ml/min/1.73 m^2^. AV fistulae were the prevailing form of vascular access (83%) and were most frequently placed by a nephrologist (61.2%). In 98% of the dialysis centers, a nephrologist was present during dialysis sessions. The following may explain the low mortality for ESRD patients in Italy: low prevalence of diabetes, high use of AV fistulae, delivery of care by nephrologists beginning in pre-ESRD stages, their involvement in placement of dialysis vascular access, and their physical presence requirement during dialysis sessions. These findings portray key aspects of the contemporary delivery of care for Italian dialysis patients and provide a platform for international comparison of healthcare systems for ESRD.

### INTRODUCTION

Approximately 1.8 million patients worldwide suffer from end-stage renal disease (ESRD) and the prevalence is increasing at an approximate rate of 7%/year[1,2,3]. Although progress is being made in renal care, particularly in CKD stages prior to renal failure[4,5], ESRD poses a significant burden to public health in terms of high mortality, morbidity, and costs[6,7]. Reasons for the high prevalence of ESRD globally include higher life expectancy of ESRD patients, increasing dialysis opportunities in countries where access had previously been limited[2,3], and an increased prevalence of diabetes[3] and the metabolic syndrome[8,9].

Mortality outcomes amid dialysis patients differ significantly internationally and within individual countries for reasons that are not completely understood[10,11,12]. As noted in the 2006 USRDS report, gross mortality was quoted by several investigators as being 24% in the U.S., 12–14% in Europe, and 9% in Japan[13]. One possibility for the differences that has been considered is the heterogeneous population mix in the U.S. as compared to the more homogeneous populations in Japan or other Western countries[13]. Demographic variations, however, explain only part of the difference in mortality both intercontinentally and within the U.S.[12]. It is possible that differences in the quality[10,14,15] and delivery of care may explain outcomes in areas such as mortality[16,17], vascular access distribution[18,19,20], and clinical outcomes[21].

The Dialysis Outcomes and Practice Patterns Study (DOPPS) has highlighted the successes of Italian treatment and outcomes in terms of fistula prevalence[7,19,22] and low mortality rates[7,16,23]. We sought to examine the Italian renal care system by means of an electronic survey in order to help identify delivery of care aspects that contribute to improved mortality outcomes in ESRD patients.

### METHODS

#### Survey of Dialysis Centers

A nationwide electronic survey, with both English and Italian versions, was sent to 575 dialysis centers with valid contact e-mail addresses (87% of all centers in Italy)[24]. The Italian Society of Nephrology (SIN) has records of the registered dialysis centers in Italy[24]. A study database was created using the SIN resources, which contained the contact information of each center, regional location, and the director of each location. Responses were received between March and June 2007, and were based strictly on prevalent data for the period of January 1, 2006 to December 31, 2006. The survey obtained official endorsement from the SIN to encourage the surveyed centers to respond.

The survey comprised 55 multiple-choice and fill-in-the blank questions distributed in the following topics: (1) center information (public, private, director's name, region); (2) ESRD specific data (hemodialysis [HD] and peritoneal dialysis [PD] patients in the center, incident patients, deceased patients, cause of ESRD); (3) demographic data and comorbid conditions (age range; number of octogenarians; prevalence of diabetes, hypertension, and cardiovascular disease); (4) aspects of diagnosis of kidney disease, such as timing (patient CKD stage at time of first nephrologist involvement; number of late referrals defined as patients referred to a nephrologist in an advanced stage of CKD fewer than 12 weeks before initiation of dialysis; frequency and method of GFR estimation) and place of diagnosis (hospital clinics, private office); (5) aspects of dialysis treatment (average estimated GFR level at first dialysis, frequency of clinical performance indicator measurements: Kt/V, Hb, albumin levels); (6) vascular access (type of vascular access, placement by nephrologist or surgeon, location and timing of access placement, maturation period for AV fistulae, maintenance practices, vascular access complications: thrombosis, infection, stenosis); (7) dialysis delivery aspects (sessions per week, hours per week, nephrologist presence).

Participants were instructed to refer to their center's medical database for 2006 to provide exact, absolute number answers to survey questions. There was no institutional review board approval needed for this study since no patient interaction occurred.

#### Regional Sampling

Fig. 1 shows the distribution of centers, the number of respondents, and the dialysis population from each of the 20 geographical regions. Based on this distribution, survey responses were stratified and weighted to reflect the actual population in each region, and to allow for results to model the Italian dialysis population as a whole. This also allowed for weighting of centers by stratum to be representative of the total number of facilities in their region.

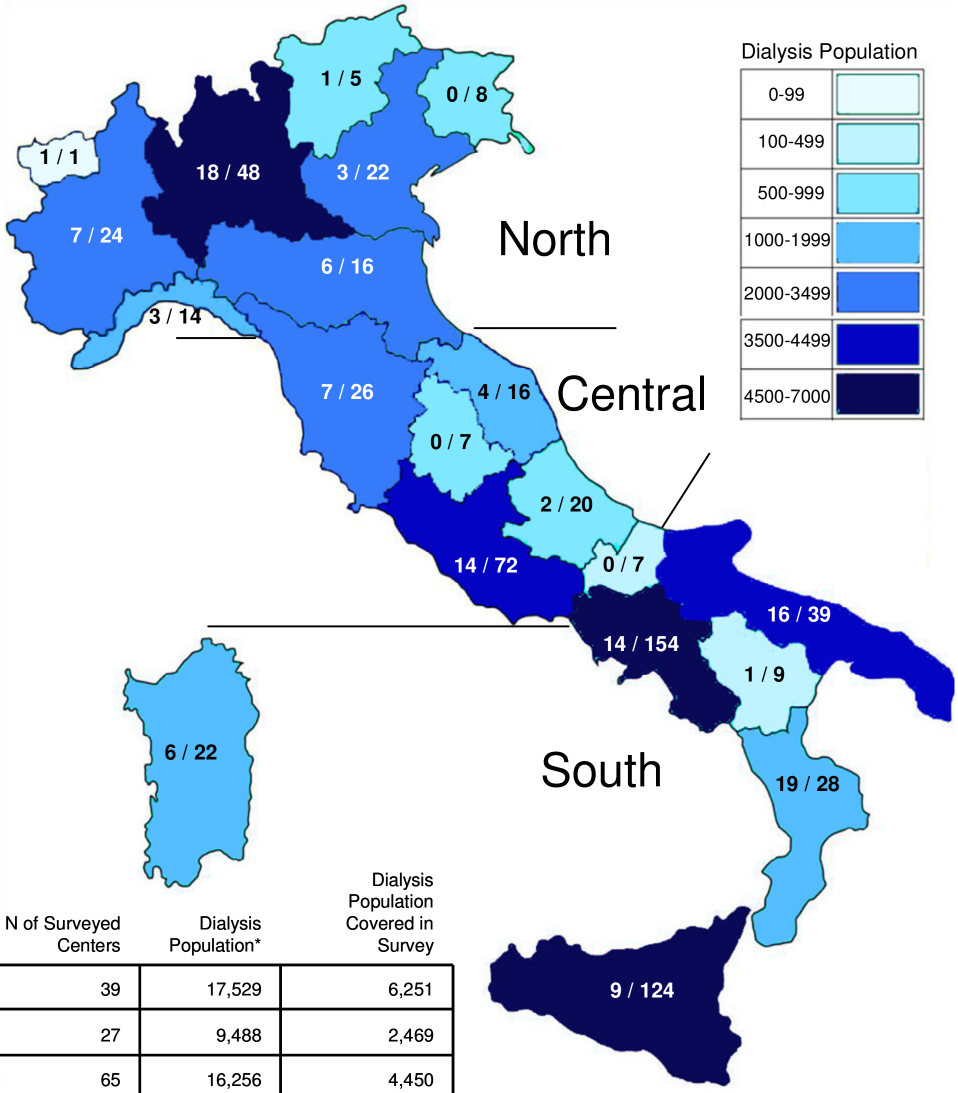

| | N of Dialysis Centers* | N of Surveyed Centers | Dialysis Population* | Dialysis Population Covered in Survey |
|---|---|---|---|---|
| North | 138 | 39 | 17,529 | 6,251 |
| Central | 148 | 27 | 9,488 | 2,469 |
| South | 376 | 65 | 16,256 | 4,450 |
| Total | 662 | 131 | 43,273 | 13,170 |

* Data from references [27-33].

Legend
The various shades of blue represent the dialysis population in the shaded region. See above for color definitions. The numbers within regions represent number of surveys received per number of centers within region (example: 6 / 22 signifies that 6 of the 22 centers located in the region responded to our survey). The table shows the actual number of dialysis centers and patients in the total ESRD population as well as the number of surveyed centers and patients.

**FIGURE 1.** Survey results by geographic region.

### Data Validation

Data from respondents were validated using quality control programs to detect errors (i.e., meet range checks, units of measure, and evaluation of nonresponse) as previously described[17,25]. All inaccurate responses from the screenings that did not correctly answer the given question (i.e., responses given in incorrect units, invalid ranges, or inaccurate number of responses) were filtered out of the study analyses. In some cases, queries produced from these quality checks were brought to the attention of primary data collectors at dialysis units in order to clarify or correct specific data components. Survey findings were compared to regional data from the Italian Dialysis and Transplantation Registry[26]. The registry has information on mortality outcomes, vascular access distribution, comorbid conditions, and primary causes of ESRD[26]. Results of the survey were in general agreement with registry data when available. Mortality data by North-Central-South regions as defined in the survey were not available for comparison.

#### Interviews

One of the authors proficient in both English and Italian (AL) conducted interviews with dialysis center directors and staff in 15 locations (see Acknowledgements) spanning 10 regions of Italy (Fig. 1). Eight interviews with centers that participated in the survey (n = 8/131) were completed in order to verify the accuracy of survey responses. Seven additional centers, which had not completed the survey, were also interviewed. The interviews provided reactions to survey results, obtained information regarding bias in selecting patients for dialysis (age and comorbid conditions), and collected opinions from nephrologists, which helped us to understand the survey results.

#### Analyses

The SPSS version 11.5 statistical package was used to weight the responses by geographical region. The regional dialysis population distribution was retrieved from data reported by the Italian Registry[27,28,29,30,31,32,33]. Comparisons among outcomes were executed by means of $\chi^2$ tests for proportions and *t* tests in addition to one-way ANOVA for means. Associations were considered significant with $p$-values < 0.05.

Gross mortality was calculated as: $\text{patients}_{\text{deceased}}/[\text{patients}_{\text{total}} + 0.5\ (\text{patients}_{\text{incident}} - \text{patients}_{\text{deceased}})]$[24,26]. This accounted for patients entering the dialysis program from January 2006 to December 2006, as well as patients previously enrolled in the program. The mortality formula used in our survey was that of the Italian Registry of Dialysis and Transplantation to allow for validation and comparison of results with the registry.

### RESULTS

#### Mortality, Patient Demographics, and Comorbidities

Of the 575 centers surveyed, 131 responded (23%), providing data from 13,170 dialysis patients (30% of the total 43,273 patients during 2006) (Fig. 1). A total of 11,645 patients were treated by HD (88%) and 1,525 patients by PD (12%). The gross mortality rate during calendar year 2006 was 11.2 ± 0.62% and included incident patients beginning in 2006.

The age range most reported was 65–75 years of age (66.7% of centers), followed by 51–65 years of age (26.6%). The other reported age ranges came from centers specialized to provide care for either pediatric (0.9% of respondents) or geriatric patients (4.1%). In response to the question of number of patients aged 80 older in each facility, we were able to compute that 14.1 ± 0.7% of all patients were octogenarians or older.

"Vascular nephropathy", a term used in Italy to refer to kidney disease attributed to hypertension and/or vascular diseases other than diabetes, was the leading cause of ESRD (31.6 ± 1.5% of centers), followed by a category of "other" (26.7 ± 1.7%), diabetes (21.0 ± 1.2%), and glomerular diseases (17.7 ± 1.2%). The category of "other" was used to identify causes of ESRD other than the aforementioned. Hypertension was the leading comorbidity (44.6 ± 2.3%), followed by cardiovascular disease (35.9 ± 2.1%), diabetes (19.4 ± 0.9%), and a combination of more than two of these comorbidities (38.4 ± 2.1%).

### Diagnosis of Kidney Disease and Dialysis Delivery

ESRD diagnosis was usually made within the setting of a hospital clinic visit (93.3% of cases) as opposed to a private physician's office visit (6.7%). At the time of referral to nephrologists, the CKD stages were stage 3 (49.8% of centers), stage 4 (22.6%), stage 2 (17.5%), and stage 5 (8.6%). Respondents did not report CKD stage 1 and 1.6% reported that CKD staging was not used. GFR was estimated in CKD stage 4 patients at the following frequencies: monthly (59.9% of respondents), bimonthly (23.6%), or every 2 weeks (7.4%). GFR was calculated using 24-h creatinine clearance (65.1% of responses), Cockcroft-Gault formula (60.8%), and eMDRD (24.6%). The percentage of late-referred patients was 27.5 ± 2.1%.

Of the respondents, 98.5% indicated that a nephrologist was always present in the center during dialysis. Patients initiated dialysis with mean estimated GFR of 9.6 ± 0.3 ml/min/1.73 $m^2$; 99% of the centers indicated that their patients received HD three times per week. The average weekly HD dose was 11.3 ± 0.2 h. The percentage of patients with Kt/V values ≥1.2 was 73.5 ± 1.8%. The percentage of patients with hemoglobin (Hb) levels ≥11 g/dl was 66.7 ± 1.8%. The percentage of the patients with albumin levels ≥4 g/dl was 50.1 ± 2.4%.

### Vascular Access

Data regarding access for dialysis are summarized in Table 1. Of note, 83% of patients were dialyzed using an AV fistula. A nephrologist completed the vascular access surgical placement alone in the majority of cases (61.2%) or in tandem with a vascular surgeon (13.0%).

**TABLE 1**
**Vascular Access**

| | | Placement of vascular access (center response %): | | | | Location (center response%) : | |
|---|---|---|---|---|---|---|---|
| | Access Type (%pts) | Nephrologist | Surgeon | Together | Other | Within dialysis center | Outside dialysis center |
| *AV Fistula* | 82.7 ± 1.0 | 61.2 | 34.7 | 13.0 | 2.0 | 52.7 | 49.8 |
| *Permanent Catheter* | 10.1 ± 0.7 | 61.0 | 24.2 | 8.9 | 11.6 | 51.3 | 51.3 |
| *AV Graft* | 4.0 ± 0.6 | 24.7 | 59.0 | 14.6 | 3.1 | 33.1 | 68.6 |
| *Temporary Catheter* | 3.2 ± 0.4 | 85.8 | 6.9 | 2.9 | 17.0 | 83.2 | 21.2 |

Of the centers, 60.9% reported thrombosis, 42.8% responded stenosis, and 4.7% reported infection as the most prevalent vascular access complications.

Access maintenance and repair for all forms of access were done by vascular surgeons alone (47.7%), interventional radiologists (36.8%), and nephrologists (29.2%). Nephrologists worked together with vascular surgeons in 20.4% of surgical operations. Total time between detecting a vascular access complication that prevented dialysis and the successive dialysis with the same vascular access averaged 52.6 ± 6.1 h. Of the respondents, 32.4% indicated that a staff and surgical facility for vascular access repair operations were available in their center.

### Regional Differences

Centers from 17 of the 20 geographic regions responded to the survey (Fig. 1). Centers from the three regions that did not respond only constitute 4% of the dialysis population[27,33]. The mortality rate in the south (9.3 ± 0.8%) and central regions (10.3 ± 0.9%) were significantly lower than in the north (15.8 ± 1.4%) ($p < 0.001$ and $p = 0.002$, respectively). There was no significant difference between the central and southern regions ($p = 0.448$) (Fig. 2).

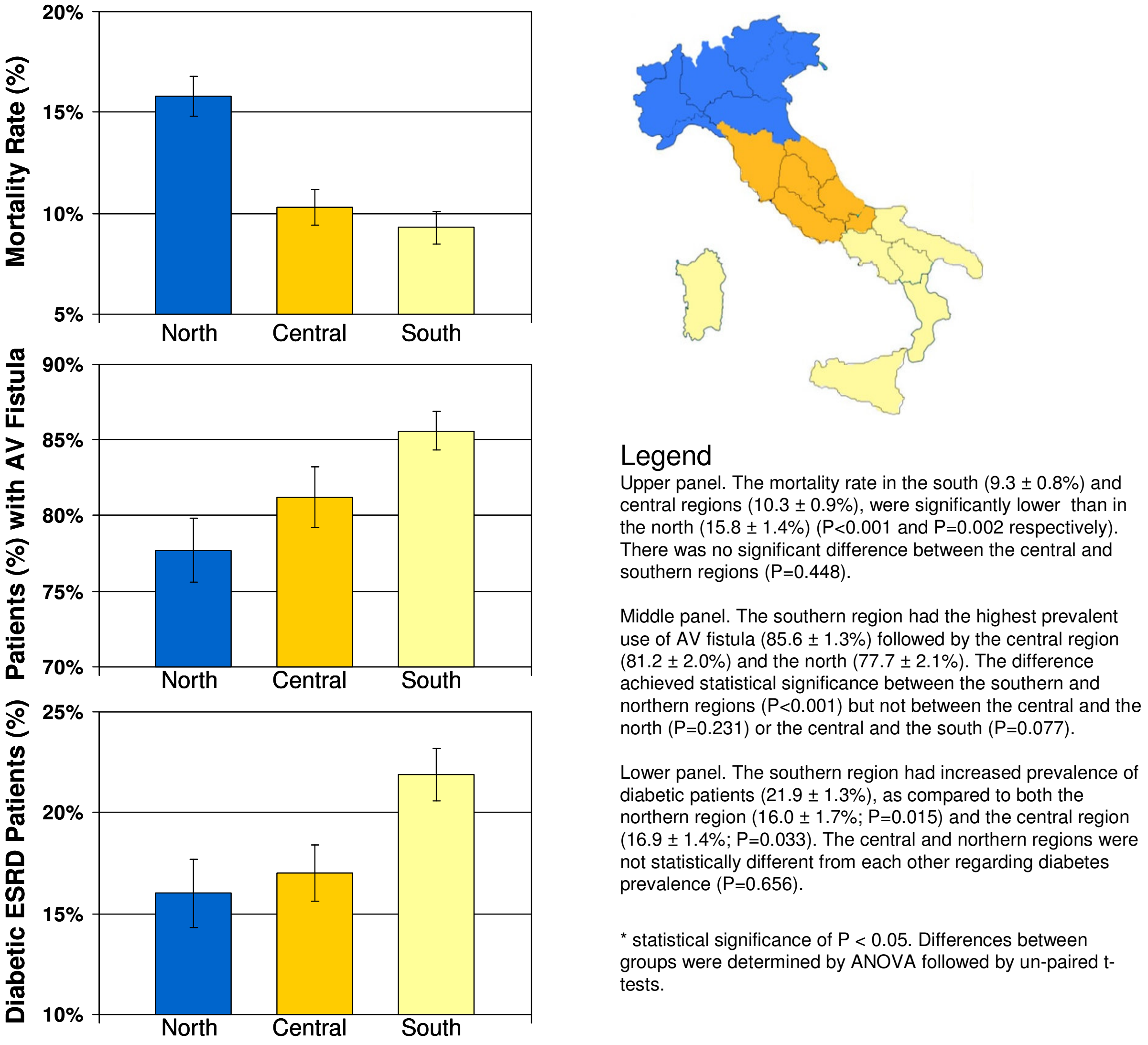

**FIGURE 2.** Geographic regional differences.

The southern region had the highest prevalent use of AV fistulae (85.6 ± 1.3%), followed by the central (81.2 ± 2.0%) and the northern (77.7 ± 2.1%) regions (Fig. 2). The difference achieved statistical significance between the southern and northern regions ($p < 0.001$), but not between the central and the north ($p = 0.231$) or the central and the south ($p = 0.077$).

The southern region had increased prevalence of diabetic patients (21.9 ± 1.3%) as compared to both the northern region (16.0 ± 1.7%; $p = 0.015$) and the central region (16.9 ± 1.4%; $p = 0.033$) (Fig. 2). The central and northern regions were not statistically different from each other regarding diabetes prevalence ($p = 0.656$).

### Public and Private Centers

Of the patients, 84% were cared for in public centers and 16% in private centers. These figures differ only slightly from the percentages of public (78%) and private (22%) centers surveyed, reflecting that public centers cared for more patients than their private counterparts; thus, our survey mainly represented patients treated in the public sector. Our survey represented the actual public and private distribution that occurs in Italy[27,28,29,30,31,32,33].

### Interviews

All nephrologists interviewed (15/15) indicated that no selection criteria exist for dialysis initiation in Italy despite age and associated comorbid conditions. All 15 interviews confirmed that nephrologists were always present during dialysis procedures and that this is mandated in Italy. Italian nephrologists stated that early referral is important in preparation for dialysis and vascular access. Interviews indicated that many Italian hospitals hold conferences between nephrologists and primary care physicians in order to recognize kidney disease progression and to allow for early referral to renal specialists.

## DISCUSSION

The low mortality rate in Italy in 2006 was markedly lower than that reported in the U.S., according to the 2006 USRDS (Table 2). The mortality derived from our survey (11.2%) is close to the 2005 Italian Renal Registry report (12.9%)[26]. The low mortality is remarkable considering that the majority of centers reported an average dialysis patient age range of either 65–75 (66.7%) or 51–65 (26.6%). These age ranges are similar to the mean age of 65 in the U.S. population[13]. Although life expectancy of the general population may be higher in Mediterranean countries than in the U.S., the difference observed in the dialysis population in both countries is well beyond any difference in the general population. When asked about any selection criteria for dialysis consideration, all interviewed nephrologists indicated that neither age nor comorbid condition prevented initiation of dialysis currently in Italy. The survey also found a significant number of octogenarians (14%) in keeping with trends in the U.S., where the number of octogenarians and nonagenarians beginning dialysis has increased considerably over the past decades[34].

While the higher mortality rate in the U.S. as compared to other Western countries is generally well appreciated[7,16,23], this survey offers insight that helps us to understand the lower mortality rate in Italy. The most striking differences between the two countries are the higher percentage of patients dialyzed with an AV fistula and the lower prevalence of diabetes in Italy as compared to the U.S. (Table 2). In the U.S., ESRD attributable to diabetes accounts for 45% of prevalent cases[13], whereas our survey estimated this in Italy to be only 19.4% (Table 2). The survey identified interesting Italian regional differences in the prevalence of diabetes that may have bearing on international mortality differences. The mortality was the highest in the northern parts of Italy, even though the reported prevalence of diabetes was lower than in southern and central regions (Fig. 2). This suggests that factors other than diabetes also importantly contribute to mortality among dialysis patients. It is possible that the higher prevalence of diabetes among dialysis patients in the U.S. as compared to Italy reflects, in part, the burden of this disease that may be more prevalent in the U.S. The nutritional status of the Italian dialysis population may also be better as suggested by the higher percentage of patients with an albumin level greater than 4.0 g/dl (Table 2).

**TABLE 2**
**Italian Survey Results Compared to U.S.**

| | USA* (%) | Italy (%) |
|---|---|---|
| *Mortality Rate* | 24.0 | 11.2 ± 0.6 |
| *AV Fistula Prevalence* | 38.6 | 82.7 ± 1.0 |
| *Diabetes Caused ESRD* | 45.0 | 21.0 ± 1.2 |
| *Diabetes Prevalence* | 44.0 | 19.4 ± 0.9 |
| *Patients with Kt/V > 1.2* | 92.8 | 73.5 ± 1.8 |
| *Patients with Hb > 11g/dL* | 87.6 | 66.7 ± 1.8 |
| *Patient with Albumin > 4.0g/dL* | 35.9 | 50.1 ± 2.4 |

* US data derived from the 2006 USRDS Annual Data Report

Our survey revealed that the use of an AV fistula as the primary form of dialysis access in Italy almost doubles that of the U.S. (Table 2). This is clearly an important difference in the delivery of care that helps to explain the lower mortality rate in Italy. The importance of AV fistulae in reducing dialysis-related infections and inflammatory burden is increasingly recognized[35]. This may lead to decreased morbidity and mortality[7,19,22]. In this respect, it is interesting to note that northern Italian regions, where the use of an AV fistula was the lowest, had the highest mortality despite a lower prevalence of diabetes. This is consistent with an assertion that increased AV fistula utilization reduces mortality even in the presence of comorbidities.

Other salient findings were (1) ESRD diagnosis made within hospital clinics, (2) 84% of patients cared for in public centers as opposed to private (16%), and (3) direct involvement of nephrologists in essentially all aspects of delivery of care including: (a) patient evaluation in CKD stages 3 and 4, (b) placement of AV fistulae, (c) vascular access maintenance in the dialysis center, and (d) mandatory presence during dialysis sessions.

The high use of AV fistulae in Italy could be traced to the direct involvement of nephrologists in the majority of vascular access management. It should be noted that there was no economic incentive to provide this service, which was done as part of the training and the traditional responsibilities of the nephrologist. Additionally, our survey revealed that Italian nephrologists were also responsible for early evaluation of CKD, vascular access maintenance, and supervision of dialysis. Dialysis initiation usually occurred when GFR was 10 ml/min/1.72 $m^2$ in keeping with NKF-K/DOQI guidelines[36]. Of note, 98.5% of respondents indicated that a nephrologist was present in the center during dialysis sessions. This physician presence requirement is common in Europe and contrasts the U.S., where nephrologists make dialysis rounds, but are not necessarily present during the dialysis procedure[37].

The mandatory presence of a nephrologist in the dialysis unit may lead to early identification of cardiovascular and infection events that may prevent unnecessary death. The detection of dialysis complications, such fistula failure, and efficient coordination of care may also be facilitated by the presence of a nephrologist. The total time between a problem that prevented dialysis on a given day and the successive session with the same vascular access averaged 52.6 h. This relatively short time interval may result from less necessity to coordinate schedules and availability between different professionals. It seems therefore reasonable to surmise that providing treatment for more aspects of vascular access by the nephrologist results in prompt and efficient delivery of care.

Another finding was that initiation of care for patients with kidney disease began within the hospital clinic system and rarely in the private physician office. While similar data are not available for the U.S. to our knowledge, it is generally known that the diagnosis of ESRD in the U.S. is often made in hospitalized patients and that many patients commence dialysis with temporary central venous catheters[22]. Early referral is known to be associated with lower morbidity[38,39] and mortality[40,41,42,43]. It is therefore possible that the lower mortality in Italy partially results from relatively early diagnosis of kidney disease (CKD stage 3).

The percentage of patients with a Kt/V ≥1.2, a measure of adequacy of dialysis, was lower in Italy than in the U.S., suggesting that the dialysis delivery dose in itself could not explain the superior mortality rate (Table 2). Our survey also found that the percentage of patients with Hb ≥11 g/dl was lower in Italy than in the U.S. The lower Hb level could be interpreted as a factor leading to either increased or reduced mortality. While Hb ≥11 g/dl has been considered generally desirable[21], recent findings of increased mortality in patients receiving erythropoietin to achieve higher levels of Hb[44] raises the possibility that the lower Hb in Italy may be another factor that helps to explain the lower mortality. Our survey was not designed to address this issue by analyzing mortality at various Hb levels[45,46,47].

The DOPPS examined differences in HD practices and outcomes internationally, with particular attention to associations with mortality risk[17,19]. Factors such as healthcare organization[7,48], comorbid conditions[12], genetic variations[10,11], and physician treatment philosophy should be considered in order to understand differences in outcomes. Our survey helps to identify possible contributions that have not been previously recognized or sufficiently emphasized. The following aspects stand out as possible explanations for the reported gross annual mortality rate (11.2%) that is about half of what is reported in the U.S.: the high use of AV fistulae as the main vascular access, the relatively low prevalence of diabetes-related renal disease, nephrological responsibility and oversight of all aspects of care starting in early stages of CKD, and nephrologist physical presence during dialysis procedures. Although the survey did not allow us to examine specifically which of the differences is most important in explaining the lower mortality, it suggests that perhaps the aggregate of these differences may account for the improved outcomes. This report highlights key aspects of the contemporary delivery of care for Italian ESRD patients and provides a model for international comparison.

### ACKNOWLEDGMENTS

We are indebted to Dr. Carmine Zoccali and the Italian Society of Nephrology for the endorsement of this project and enthusiastic support, as well as logistic aid to make this study possible.

We would like to specially thank the following nephrologists for participating in interviews: S. Boldina (Ancona); G. Mingardi (Bergamo); G. Cancarini, G. Brunori, R. Zubani (Brescia); P. Altieri (Cagliari), C. Grillo, G. Bonforte, S. Mangano, D. Martinelli (Como); F. Pizzarella (Florence); T. Zattera (La Spezia); E. Buongiorno (Lecce); G. Bacchini (Lecco); F. Casino, A. Lopez (Matera); P. Messa (Milan); D. Salvatore, C. Buzio (Parma); C. Stefano, N. Di Daniele (Rome); P. Marchini (Venice); and N. Tessitore (Verona).

The following nephrologists contributed suggestions and helped to formulate the electronic questionnaire: G. Mingardi, G. Bacchini, M. Galleini, and A. Giordani.

We extend a special thanks to Dr. G. Bacchini for his collaboration in allowing observation of vascular access surgical procedures and support of the project at the SIN first annual Vascular Access Conference.

We thank Dr. Gerald Beathard, Terry Litchfield, Richard Nee, and SonBinh T. Nguyen, Ph.D., for their advice and support to Alexander Lauder while applying for a Fulbright Research Fellowship.

We extend a sincere thanks to Scott P. Lauder, Ph.D., for his aid in the statistical analyses for this report.

A Fulbright Research Fellowship provided funding to Alexander Lauder for a 12-month stay in Bergamo, Italy for study design, collection, management, and analysis. This study was conceived as a part of a collaboration program between Northwestern University's Feinberg School of Medicine Division of

Nephrology/Hypertension and the Mario Negri Research Institute (*Istituto di Ricerche Farmacologiche «Mario Negri»*).

## References

1. Lysaght, M.J. (2002) Dialysis population dynamics: current trends and long-term implications. *J. Am. Soc. Nephrol.* **13,** S37–S40.
2. Grassmann, A., Gioberge, S., Moeller, S., and Brown, G. (2005) ESRD patients in 2004: global overview of patient numbers, treatment modalities and associated trends. *Nephrol. Dial. Transplant.* **20,** 2587–2593.
3. El Nahas, A.M. and Bello, A.K. (2005) Chronic kidney disease: the global challenge. *Lancet* **365,** 331–340.
4. Batlle, D., Ramadugu, P., and Soler, M.J. (2006) Progress in retarding the progression of advanced chronic kidney disease: grounds for optimism. *Kidney Int.* **70,** S40–S44.
5. Ruggenenti, P. and Remuzzi, G. (2007) Kidney failure stabilizes after a two-decade increase: impact on global (renal and cardiovascular) health. *Clin. J. Am. Soc. Nephrol.* **2,** 591–596.
6. National Kidney Foundation (2002) K/DOQI clinical practice guidelines for chronic kidney disease: definition and classification of stages of chronic kidney disease. *Am. J. Kidney Dis.* **39,** S1–S40.
7. Dor, A., Pauly, M.V., Eichleay, M.A., and Held, P.J. (2007) End-stage renal disease and economic incentives: the International Study of Health Care Organization and Financing (ISHCOF). *Int. J. Health Care Finance Econ.* **7(2–3),** 73–111.
8. Cameron, A., Shaw, J., and Zimmet, P. (2004) The metabolic syndrome: prevalence in worldwide populations. *Endocrinol. Metab. Clin. North Am.* **33,** 351–375.
9. Ford, E.S., Giles, W.H., and Dietz, W.H. (20070 Prevalence of the metabolic syndrome among US adults: findings from the Third National Health and Nutrition Examination Survey. *JAMA* **287,** 356–359.
10. Yoshino, M., Kuhlmann, M.K., Kotanko, P., et al. (2006) International differences in dialysis mortality reflect background general population atherosclerotic cardiovascular mortality. *J. Am. Soc. Nephrol.* **17,** 3510–3519.
11. Van Dijk, P.C.W., Zwinderman, A.H., Dekker, F.W., et al. (2007) Effect of general population mortality on the north–south mortality gradient in patients on replacement therapy in Europe. *Kidney Int.* **71,** 53–59.
12. Goodkin, D.A., Bragg-Gresham, J.L., Koenig, K.G., et al. (2003) Association of comorbid conditions and mortality in hemodialysis patients in Europe, Japan, and the United States: the Dialysis Outcomes and Practice Patterns Study (DOPPS). *J. Am. Soc. Nephrol.* **14,** 3270–3277.
13. United States Renal Data System (2006) USRDS 2006 Annual Data Report. National Institutes of Health, National Institute of Diabetes and Digestive and Kidney Diseases.
14. Held, P.J., Blagg, C.R., Liska, D.W., Port, F.K., Hakim, R., and Levin, N. (1992) The dose of hemodialysis according to dialysis prescription in Europe and the United States. *Kidney Int.* **38,** S16–S21.
15. Kjellstrand, C. and Blagg, C.R. (2003) Differences in dialysis practice are the main reasons for the high mortality rate in the United States compared to Japan. *Hemodial. Int.* **7,** 59–66.
16. Rayner, H.C., Pisoni, R.L., Bommer, J., et al. (2004) Mortality and hospitalization in haemodialysis patients in five European countries: results from the Dialysis Outcomes and Practice Patterns Study (DOPPS). *Nephrol. Dial. Transplant.* **19,** 108–120.
17. Young, E.W., Goodkin, D.A., Mapes, D.L., et al. (2000) The Dialysis Outcomes and Practice Patterns Study (DOPPS): an international hemodialysis study. *Kidney Int.* **57,** S74–S81.
18. Bonucchi, D., D'Amelio, A., Capelli, G., and Albertazzi, A. (1999) Management of vascular access for dialysis: an Italian survey. *Dial. Transplant. News* **14,** 2116–2118.
19. Rayner, H.C., Besarab, A., Brown, W.W., Disney, A., Saito, A., and Pisoni, R.L. (2004) Vascular access results from the Dialysis Outcomes and Practice Patterns Study (DOPPS). *Am. J. Kidney Dis.* **44,** S22–S26.
20. Besarab, A. (2002) Preventing vascular access dysfunction: which policy to follow. *Blood Purif.* **20,** 26–35.
21. Rocco, M.V., Frankenfield, D.L., Hopson, S.D., and McClellan, W.M. (2006) Relationship between clinical performance measures and outcomes among patients receiving long-term hemodialysis. *Ann. Intern. Med.* **145,** 512–519.
22. Pisoni, R.L., Young, E.W., Dykstra, D.M., et al. (2002) Vascular access use in Europe and the United States: results from the DOPPS. *Kidney Int.* **61,** 305–316.
23. Goodkin, D.A., Young, E.W., Kurokawa, K., Prutz, K.G., and Levin, N.W. (2004) Mortality among hemodialysis patients in Europe, Japan, and the United States: case-mix effects. *Am. J. Kidney Dis.* **44,** S16–S21.
24. Società Italiana di Nefrologia. Italian Society of Nephrology. www.sin-italy.org 2007.
25. Pisoni, R.L., Gillespie, B.W., Dickinson, D.M., Chen, K., Kutner, M.H., and Wolfe, R.A. (2004) The Dialysis Outcomes and Practice Patterns Study (DOPPS): design, data elements, and methodology. *Am. J. Kidney Dis.* **44,** S7–S15.
26. Conte, F. (2007) Censimento dei Centri di Nefrologia e Dialisi italiani, Risultati 2005.
27. Simeoni, P.G., Bonomini, M., Brigante, M., et al. (2006) Censimento 2004 Centri di Nefrologia e Dialisi Italiani. Abruzzo – Lazio – Marche – Molise – Umbria. *G. Ital. Nefrol.* **1,** 64–75.

28. Gesualdo, L., Alloatti, S., Cicchetti, T., et al. (2006) Censimento 2004 Centri di Nefrologia e Dialisi Italiani. Basilicata – Calabria – Puglia. *G. Ital. Nefrol.* **3,** 323–336.
29. Alloatti, S., Daidone, G., Avella, F., and Cogoni, G. (2006) Censimento 2004 Centri di Nefrologia e Dialisi Italiani. Campania – Sicilia – Sardegna. *G. Ital. Nefrol.* **3,** 337–349.
30. Buccianti, G., Alloatti, S., Conte, F., and Pedrini, L. (2006) Censimento 2004 Centri di Nefrologia e Dialisi Italiani. Confronto tra Piemonte e Lombardia. *G. Ital. Nefrol.* **2,** 193–202.
31. Lusenti, T., Santoro, A., Cappelli, G., et al. (2006) Censimento 2004 Centri di Nefrologia e Dialisi Italiani. Emilia-Romagna – Toscana. *G. Ital. Nefrol.* **2,** 203–211.
32. Alloatti, S., Garibotto, G., Triolo, G., Quarello, F., Salomone, M., and Buccianti, G. (2005) Censimento 2004 Centri di Nefrologia e Dialisi Italiani. Piemonte – Liguria – Valle d'Aosta. *G. Ital. Nefrol.* **4,** 354–364.
33. Nordio, M., Laudon, A., Panzetta, G.O., Marchini, P., and Alloatti, S. (2006) Censimento 2004 Centri di Nefrologia e Dialisi Italiani. Veneto – Friuli Venezia Giulia – Trentino Alto Adige. *G. Ital. Nefrol.* **2,** 182–192.
34. Kurella, M., Covinsky, K.E., Collins, A.J., and Chertow, G.M. (2007) Octogenarians and nonagenarians starting dialysis in the United States. *Ann. Intern. Med.* **146,** 177–183.
35. Stevenson, K.B., Hannah, E.L., Lowder, C.A., et al. (2002) Epidemiology of hemodialysis vascular access infections from longitudinal infection surveillance data: predicting the impact of NKF-DOQI clinical practice guidelines for vascular access. *Am. J. Kidney Dis.* **39,** 549–555.
36. National Kidney Foundation (2001) K/DOQI clinical practice guidelines for hemodialysis adequacy: update 2000. *Am. J. Kidney Dis.* **37,** S7–S64.
37. Mentari, A.K., DeOreo, P.B., O'Connor, A.S., Love, T.E., Ricanati, E.S., and Sehgal, A.R. (2005) Changes in Medicare reimbursement and patient-nephrologist visits, quality of care, and health-related quality of life. *Am. J. Kidney Dis.* **46,** 621–627.
38. Gallego, E., López, A., Lorenzo, I., et al. (2003) Influence of early or late referral to nephrologist over morbidity and mortality in hemodialysis. *Nefrologia* **23,** 234–242.
39. Jungers, P., Zingraff, J., Albouze, G., et al. (1993) Late referral to maintenance dialysis: detrimental consequences. *Nephrol. Dial. Transplant.* **8,** 1089–1093.
40. Khan, I.H., Catto, G.R., Neil, E., and Macleod, A.M. (1995) Death during the first 90 days of dialysis: a case-control study. *Am. J. Kidney Dis.* **25,** 275–280.
41. Locatelli, F., Del Vecchio, L., and Pozzoni, P. (2002) The importance of early detection of chronic kidney disease. *Nephrol. Dial. Transplant.* **17,** S2–S7.
42. Roubicek, C., Brunet, P., Huiart, L., et al. (2000) Timing of nephrology referral: influence on mortality and morbidity. *Am. J. Kidney Dis.* **36,** 35–41.
43. Lin, C.L., Chuang, F.R., Wu, C.F., and Yang, C.T. (2004) Early referral as an independent predictor of clinical outcome in end-stage renal disease on hemodialysis and continuous ambulatory peritoneal dialysis. *Ren. Fail.* **26,** 537–537.
44. Eckardt, K.U. (2001) Target hemoglobin in patients with renal failure. *Nephron* **89,** 135–144.
45. Besarab, A., Bolton, W.K., Browne, J.K., et al. (1998) The effects of normal as compared with low hematocrit values in patients with cardiac disease who are receiving hemodialysis and epoetin. *N. Engl. J. Med.* **339(9),** 584–590.
46. Singh, A.K., Szczech, L., Tang, K.L., et al. (2006) Correction of anemia with epoetin alfa in chronic kidney disease. *N. Engl. J. Med.* **355(20),** 2085–2098.
47. Drueke, T.B., Locatelli, F., Clyne, N., et al. (2006) Normalization of hemoglobin level in patients with chronic kidney disease and anemia. *N. Engl. J. Med.* **355(20),** 2071–2084.
48. Locatelli, F., Pontoriero, G., Del Vecchio, L., and Conte, F. (2000) Technical-economic management models in dialytic treatment: an evolving reality. The Italian experience. *J. Nephrol.* **13,** S20–S27.
